# A multi-omic resource of wheat seed tissues for nutrient deposition and improvement for human health

**DOI:** 10.1038/s41597-023-02133-y

**Published:** 2023-05-10

**Authors:** Jingjing Zhi, Jian Zeng, Yaqiong Wang, Hongyan Zhao, Guoli Wang, Jing Guo, Yuesheng Wang, Mingjie Chen, Guangxiao Yang, Guangyuan He, Xiaoyuan Chen, Junli Chang, Yin Li

**Affiliations:** 1 The Genetic Engineering International Cooperation Base of Chinese Ministry of Science and Technology, The Key Laboratory of Molecular Biophysics of Chinese Ministry of Education, College of Life Science and Technology, Huazhong University of Science & Technology, Wuhan, 430074 China; 2 Guangdong Provincial Key Laboratory of Utilization and Conservation of Food and Medicinal Resources in Northern Region, Henry Fok School of Biology and Agriculture, Shaoguan University, Shaoguan, Guangdong 512005 China

**Keywords:** Plant physiology, Secondary metabolism, Plant molecular biology

## Abstract

As a globally important staple crop, wheat seeds provide us with nutrients and proteins. The trend of healthy dietary has become popular recently, emphasizing the consumption of whole-grain wheat products and the dietary benefits. However, the dynamic changes in nutritional profiles of different wheat seed regions (*i.e*., the embryo, endosperm and outer layers) during developmental stages and the molecular regulation have not been well studied. Here, we provide this multi-omic resource of wheat seeds and describe the generation, technical assessment and preliminary analyses. This resource includes a time-series RNA-seq dataset of the embryo, endosperm and outer layers of wheat seeds and their corresponding metabolomic dataset, covering the middle and late stages of seed development. Our RNA-seq experiments profile the expression of 63,708 genes, while the metabolomic data includes the abundance of 984 metabolites. We believe that this was the first reported transcriptome and metabolome dataset of wheat seeds that helps understand the molecular regulation of the deposition of beneficial nutrients and hence improvements for nutritional and processing quality traits.

## Background & Summary

Wheat is one of the “big three” cereals that dominate global staple food production. Wheat seeds are milled to remove brans, leaving most starchy endosperm to produce refined white flour, which are mainly starch and proteins. Wheat has become such a popular staple crop probably because of two major reasons. First, wheat is one of the most important sources of total calorie intake and contributes significantly to our daily plant-sourced proteins, fibres, mineral nutrients and beneficial phytochemicals (*i.e*., bioactive compounds)^1^. Second, wheat seeds contain a unique set of seed storage proteins (*i.e*., glutenins and gliadins), which form polymeric protein networks as the physico-chemical basis of unique visco-elastic properties, allowing wheat dough made into numerous types of flour foods consumed globally, such as breads.

High yield and environmental adaptability have long been the main targets of wheat breeding^2^. In recent years, the conceptual changes of “eat full” to “eat well” have become popular in food consumption, emphasizing the nutrition value and dietary benefits of our daily meal. Nutritional studies provide evidence that long-term intake of refined white-flour foods (*e.g*., white flour breads) as the staple is not beneficial for human health and is associated with the prevalence and development of chronic diseases, such as cardiovascular diseases and type-2 diabetes^1^. By contrast, consumption of wholegrain wheat-based food products has become the new dietary trend, as wheat bran provides many health-beneficial compounds^3^. Thereafter, increasing efforts have been made in the basic and applied research areas to understand the genetic basis of wheat quality, especially the nutritional quality traits, and to genetically improve these traits^4,5^. For example, several key enzymes in the carotenoid biosynthetic pathway have been functionally studied, including β-hydroxylase (HYD)^6^, lycopene epsilon cyclase (LCYε)^7^, carotenoid cleavage dioxygenases (CCD)^8^ and aldehyde oxidase (AO)^9,10^, and transgenic wheat lines or mutant lines have been investigated to discover their effects on biofortification of β-carotene in wheat^11–15^. More recently, owing to the technological advances in metabolomics (*e.g*., the widely targeted metabolomics based on ultra-performance liquid chromatography- electrospray ionization-tandem mass spectrometry (UPLC-ESI-MS/MS)^16^), large-scale identification and quantification of phytochemicals in wheat seeds has become possible. Thus, the metabolomes of a few wheat cultivars with black, purple or blue grain colors have been studied, revealing the metabolic basis of grain color formation and possible molecular mechanisms^17–21^.

Based on the above-mentioned studies, it is demonstrated that: (1) the human-beneficial phytochemicals are deposited in wheat seeds in a spatial and temporal manner; (2) pigmented wheat varieties offer high levels of bioactive compounds (flavonoids, phenolics, vanillin, and azelaic acid, for instance). Many of the bioactive compounds are accumulated in the outer layers of wheat seeds and their contents vary between wheat varieties, which not only serve as the basis supporting the benefits of whole-grain wheat consumption, but, unfortunately, are mostly removed during the grain milling process^22^. Our group previously showed that the contents of major carotenoids in wheat (lutein, zeaxanthin and β-carotene) were drastically decreased during seed development, possibly representing an intrinsic limitation in carotenoid biofortification of wheat seeds^14^.

Similar to the phytochemical deposition in seeds, the seed storage proteins (SSPs) of wheat are accumulated in spatial-temporal patterns as well. These SSPs include a large portion of gluten proteins, such as high-molecular-weight glutenin subunit (HMW-GS), low-molecular-weight glutenin subunit (LMW-GS), gliadins, puroindolines and avenin-like proteins (ALPs)^23^. The expression of these SSP-encoding genes are dramatically up-regulated from 12 to 20 days after pollination (DAP) and peak at the middle and late stages of endosperm development (20~30 DAP)^24–26^. In addition, these SSPs are accumulated in different seed regions: HMW-GS and γ-gliadin are primarily deposited in the central endosperm cells, while the S-rich prolamins (LMW-GS, α-gliadin and ALPs) are abundant in the sub-aleurone layer and the adjacent cells^27–29^. The spatial-temporal expression of SSP-encoding genes are tightly controlled by a combination of different transcription factors, some of which have been functionally characterized with recent advancements in wheat genomics and genetics^28,30–33^.

In the well-studied Poaceae species (rice and maize), complex networks of transcription factors and coregulators are known to be involved in the regulation of storage proteins and accumulation of starch and other phytochemicals, serving as, at least partly, the genetic basis of yield and nutritional and processing quality traits^34,35^. Compared to rice and maize, the post-genomic era of wheat studies has come until the very recent burst of Triticeae genomic resources, landmarking by the release of high-quality reference genomes of common wheat, durum wheat and their diploid ancestors^36–41^. While the recent accumulation of Triticeae genomic datasets, high quality transcriptomic and metabolomic resources of wheat seeds with focus on the spatial-temporal regulation of proteins and phytochemicals are scarce. The expression atlas of wheat includes a few samples of aleurone and endosperm^42^, while the other public RNA-seq datasets of wheat seeds emphasize on embryogenesis^43^. Also, environmental factors are known to contribute to grain development and metabolite abundance^19,44^, suggesting the necessity of a well-designed field experiment in generating transcriptome and metabolome datasets simultaneously for studying seed development. In the present work, we describe a high-quality, 310-GB RNA-seq data of wheat seed tissues together with a metabolomic dataset produced from the same samples. This multi-omic resource covers the stages and tissues suitable for studying the nutrient deposition and protein accumulation during late grain development. We believe that this resource will be helpful for understanding the spatial-temporal patterns of seed proteins and beneficial phytochemicals, for unraveling the regulatory networks involved in these metabolic processes and for wheat quality improvement.

## Methods

### Plant materials and field experiments

**Fig. 1:**
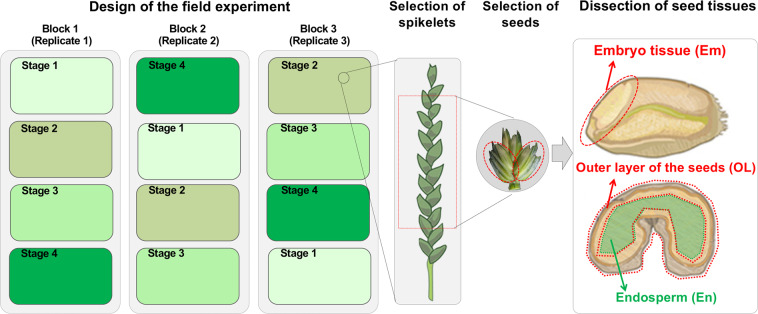
Overview of the experimental design and sample collection for RNA-seq and metabolomics.

The bread wheat (*Triticum aestivum* L.) cultivar L88-31 was used in the field experiment, which was conducted at the experimental field of Huazhong University of Science and Technology in Wuhan, China during the 2020~2021 season using a randomized completely block design with three replicates (Fig. 1). L88-31 belongs to a set of wheat near isogenic lines that have been significantly contributed to our understanding on wheat dough property and widely used for producing transgenic wheat lines^45–47^. Each block was used to collect the samples of four stages for a biological replicate and the sampling stage was randomly assigned to a plot within each block. Each plot consisted of twelve rows which were 1.5 m long and had 35 seeds per row. Since the focus of our research is to capture the dynamics of gene expression and metabolites during the late stages of wheat seed development, the four stages were used: 20, 25, 30 and 35 days after pollination (DAP). Within each plot, the main tillers and the corresponding spikes were tagged and used for sample collection. The middle part of each sampled spikes was used for seed collection and dissection of tissue samples to avoid samples from different flowering time and seed developmental status. All the spikes were collected in the morning (between 9:00 A.M. to 11:00 A.M.) to avoid potential influences of circadian on transcriptome and metabolome. Once collected from the field, the spikes were transferred to the lab and dissected on ice with scalpels and tweezers immediately to embryos, the out layer of seeds and endosperm tissues (abbreviated as Em, OL, and En, respectively, and used hereafter), followed by snap frozen in liquid nitrogen. Around 150~200 seeds from the same plot were sampled and pooled together to form a biological replicate (including the tissues for both RNA-seq and metabolomics) (Fig. 1). Owing to the tissue availability, the embryo tissues collected at 20, 25, 30, and 35 DAP (Em20, Em25, Em30, and Em35, respectively) were used for RNA-seq analysis and other experiments in future, while the endosperm and outer layer tissues collected at 20, 25, 30, and 35 DAP (En20, En25, En30, En35, and OL20, OL25, OL30, OL35, respectively) were used for both metabolomics and RNA-seq, with the remaining tissues saved for future experiments.

### RNA extraction, library construction and sequencing

Total RNA was extracted with TRIzol reagent. The quality of extracted RNA samples was examined by agarose gel electrophoresis, NanoDrop 2000, and Agilent 2100 Bio-analyzer (Table 1). Standard protocols for the Illumina NovaSeq platform were used for construction of the wheat mRNA libraries. RNA-seq libraries were sequenced to generate 150-bp pair-end reads. For sequence quality control, cutadapt (https://cutadapt.readthedocs.io/en/stable/)^48^ and FASTX-Toolkit (http://hannonlab.cshl.edu/ fastx_toolkit/) were used to trim low-quality base pairs from the 3′ end of each sequence and the quality of raw and clean data was checked with FastQC (https://www.bioinformatics.babraham.ac.uk/projects/fastqc/). The quality-filtered clean reads were mapped to the wheat reference genome of Chinese Spring (IWGSC_v1.0) using HISAT2 v2.0.1-beta with default parameters (https://daehwankimlab.github.io/hisat2/)^39,49^. Only uniquely-mapped reads were retained and the read counts that aligned to the 110,790 gene models annotated of the wheat reference genome were calculated by using the featureCount software (https://subread.sourceforge.net/featureCounts.html)^50^. FPKM (fragments per kilobase of exon per million mapped sequence reads) values were calculated for each gene model. Genes met the following criteria were considered as expressed in a stage: (1) at least 5 reads mapped to a gene in each of the three replicates; (2) the average FPKM at a stage should be ≥ 0.5. The Pearson correlation coefficients between biological replicates were calculated using gene expression values. The differentially expressed genes (DEGs) were determined with edgeR by comparing the read count data between stages within each tissue or by comparing the tissues of each stages (https://bioconductor.org/packages/release/bioc/html/edgeR.html)^51,52^. The significance threshold for edgeR-based differentially expressed genes was fold change ≥ 2 and a false discovery rate (FDR)-adjusted *P*-value < 0.05.

**Table 1:** The quality of each RNA sample. RIN, RNA integrity.

Sample Name	RNA Conc.	volume	quantity	A260/280	A260/230	RIN
En20-1	342	35	11.97	1.92	1.64	8.7
En20-2	404	35	14.14	1.88	1.51	8.4
En20-3	444	35	15.54	1.92	1.75	8.6
En25-1	356	35	12.46	1.87	1.32	8.7
En25-2	165	35	5.78	1.93	1.68	8.5
En25-3	252	35	8.82	1.94	1.56	8.6
En30-1	228	35	7.98	1.93	1.75	8.8
En30-2	304	35	10.64	1.92	1.67	8.2
En30-3	196	35	6.86	1.94	1.71	8.4
En35-1	118	35	4.13	1.94	1.72	8.6
En35-2	230	35	8.05	1.96	1.74	8.7
En35-3	218	35	7.63	1.93	1.68	8.3
OL20-1	478	35	16.73	1.91	1.72	8.8
OL20-2	421	35	14.74	1.94	1.74	8.9
OL20-3	387	35	13.55	1.93	1.76	8.7
OL25-1	365	35	12.78	1.95	1.68	8.9
OL25-2	358	35	12.53	1.92	1.62	8.5
OL25-3	401	35	14.04	1.96	1.63	8.6
OL30-1	369	35	12.92	1.92	1.71	8.4
OL30-2	374	35	13.09	1.93	1.68	8.7
OL30-3	362	35	12.67	1.94	1.74	8.6
OL35-1	241	35	8.44	1.92	1.62	8.8
OL35-2	231	35	8.09	1.96	1.68	8.7
OL35-3	242	35	8.47	1.91	1.72	8.4
Em20-1	231	35	8.09	1.91	1.72	8.1
Em20-2	189	35	6.62	1.94	1.74	8.0
Em20-3	187	35	6.55	1.93	1.76	7.7
Em25-1	265	35	9.28	1.95	1.68	7.9
Em25-2	158	35	5.53	1.92	1.62	7.5
Em25-3	201	35	7.04	1.96	1.63	7.6
Em30-1	269	35	9.42	1.92	1.71	8.3
Em30-2	174	35	6.09	1.93	1.68	7.7
Em30-3	162	35	5.67	1.94	1.74	7.6
Em35-1	241	35	8.44	1.92	1.62	7.8
Em35-2	231	35	8.09	1.96	1.68	8.7
Em35-3	182	35	6.37	1.91	1.72	8.4

### Metabolomic analysis

To capture the metabolomic changes during wheat seed development, quasi-targeted metabolomics was employed to analyze the wheat samples under a contract with Novogene Co., Ltd. (Beijing, China). The metabolomic method was established by Novogene and reported previously^53–55^. The method is described in detail as below.

#### Metabolites extraction

Wheat tissues (100 mg per sample) were grounded individually into powder with liquid nitrogen and the homogenate was well vortexed together with prechilled 500 μL 80% methanol. The samples were then subject to a 5-min cold incubation (on ice) followed by centrifugation (15,000 g, 4 °C for 20 min). The supernatant was diluted with LC-MS grade water to a final concentration of 53% methanol. The samples were subsequently transferred to a new Eppendorf tube followed by centrifugation (15,000 g, 4 °C for 20 min). After that, the supernatant was injected into the LC-MS/MS system^56,57^.

#### HPLC-MS/MS analysis

LC-MS/MS analyses were conducted with an ExionLC™ AD system (SCIEX) coupled with a QTRAP® 6500+ mass spectrometer (SCIEX). By using a 20-min linear gradient at a flow rate of 0.4 mL/min for the positive/negative polarity mode, the sample was injected onto a Xselect HSS T3 (2.1 × 150 mm, 2.5 μm) with 0.1% formic acid-water and 0.1% formic acid-acetonitrile as the eluent A and B, respectively^54^. The following parameters for solvent gradient was used: 2% B, 2 min; 2–100% B, 15.0 min; 100% B, 17.0 min; 100-2% B, 17.1 min; 2% B, 20 min. QTRAP® 6500+ mass spectrometer was operated in the positive polarity mode with curtain gas of 35 psi, collision gas of medium, ionspray voltage of 5500 V, temperature of 550 °C, ion source gas of 1:60, and ionsource gas of 2:60. QTRAP® 6500+ mass spectrometer was operated in the negative polarity mode with the following settings: curtain gas of 35 psi, collision gas of medium, ionspray voltage of −4500 V, temperature of 550 °C, ion source gas of 1:60, ion source gas of 2:60.

#### Metabolites identification and quantification

Metabolite identification was based on the in-house database using MRM (Multiple Reaction Monitoring)^58^. In the in-house library of Novogene Co., Ltd., more than 2500 commercially available purified standard compounds have been registered to the LC-MS/MS platform for determination of their characteristics. This in-house database includes 190+ amino acids and derivatives, 200+ organic acids and derivatives, 100+ nucleotide and derivatives, more than 120 flavonoid compounds, more than 40 anthocyanins, over 30 vitamin compounds, 30+ alkaloids and derivatives, 20+ phenolamides, 95+ carbohydrates and derivatives, 100+ lipids (including phospholipids, fatty acyls, glycerophospholipids and glycerolipids), 20+ phytohormone compounds, 600+ known compounds from medicinal plants and over 1000 other compounds (such as anthracenes, benzene and substituted derivatives, cinnamic acids and derivatives, coumarins and derivatives, iridoid derivatives, etc.).

To accurately identify biochemicals, retention time (RT) with a narrow RT window, accurate mass match to the library entries (+/− 0.005 amu), Q1 (parent ion) and Q3 and the MS/MS forward and reverse scores between the experimental data and the authentic standards were applied as the criteria^59,60^. SCIEX OS (version 1.4) was used to process the HPLC-MS/MS data files to integrate and correct the peak with the following parameters: minimum peak height, 500; signal/noise ratio, 5; gaussian smooth width, 1. The area of each peak represents the relative content of the corresponding substance.

#### Metabolomic data analysis

These identified metabolites were annotated using, Human Metabolome Database (HMDB) database^61^, Lipidmaps database^62^ and the Kyoto Encyclopedia of Genes and Genomes (KEGG) database^63^. Principal components analysis (PCA) and partial least squares discriminant analysis (PLS-DA) were conducted with the metabolomics software metaX^64^. We applied univariate analysis (*t*-test) to calculate the statistical significance (*P*-value). The following criteria was used to identify differential metabolites: variable important in projection value VIP > 1 and *P*-value < 0.05, log2(fold change) ≥ 1 or ≤ −1.

## Data Records

This data set contains two parts: the first part is the RNA-seq data and the derived gene expression data; the second part is the results of metabolite identification and quantification with metabolomics. Both parts use the same set of sample names, in which En, Em and OL stands for the endosperm, embryo, and outer layer tissues, respectively, with two digits standing for seed developmental stages and the suffix standing for biological replicates. For the RNA-seq data, the raw sequencing data containing all 36 samples were deposited in the NCBI (Bioproject ID: PRJNA891918; SRA experiments No. SRP430408)^65^. The gene expression matrix and the results of differential expression analysis are available at figshare^66^. For the metabolomic data, the metabolite information (*i.e*., compound name, formula, exact Q1 (m/z), molecular weight, RT and CAS No.), relative quantities and differential metabolite results have been made available by Novogene Co., Ltd. and are deposited at figshare^67,68^. Owing to the contract with Novogene, the raw metabolomic data have not been publicly available. Other data related to this data set (such as RNA quality, RNA-seq statistics) are provided in the manuscript (Table 1, Supplementary Tables S1–S3).

## Technical Validation

### Quality control

The quality of extracted total RNA is key to high-quality RNA-seq libraries and successful downstream experiments, because low RNA integrity (RIN) values may affect the quality of RNA-seq libraries and may lead to potential deviations in gene expression. In our project, we ensured that the RIN values of all RNA samples were > 7.0. The quality parameters of each RNA samples are shown in Table 1.

## Quality Validation

### Quality validation for the RNA-seq samples

To ensure that the transcriptome and metabolome data obtained meet statistical standards, we have designed the experiment in consideration of several potential influencing factors, including replicates of the fields, growth differences between major tillers and smaller, minor tillers, differences in the developmental rate between the seeds from upper, middle and lower parts of the spike, and growth differences between individual plants and seeds (Fig. 1). In our experiments, we sampled several hundreds of seeds for each sample and the seeds were dissected on ice into three tissues: embryo (Em), the out layers (OL) and endosperm (En).

**Fig. 2:**
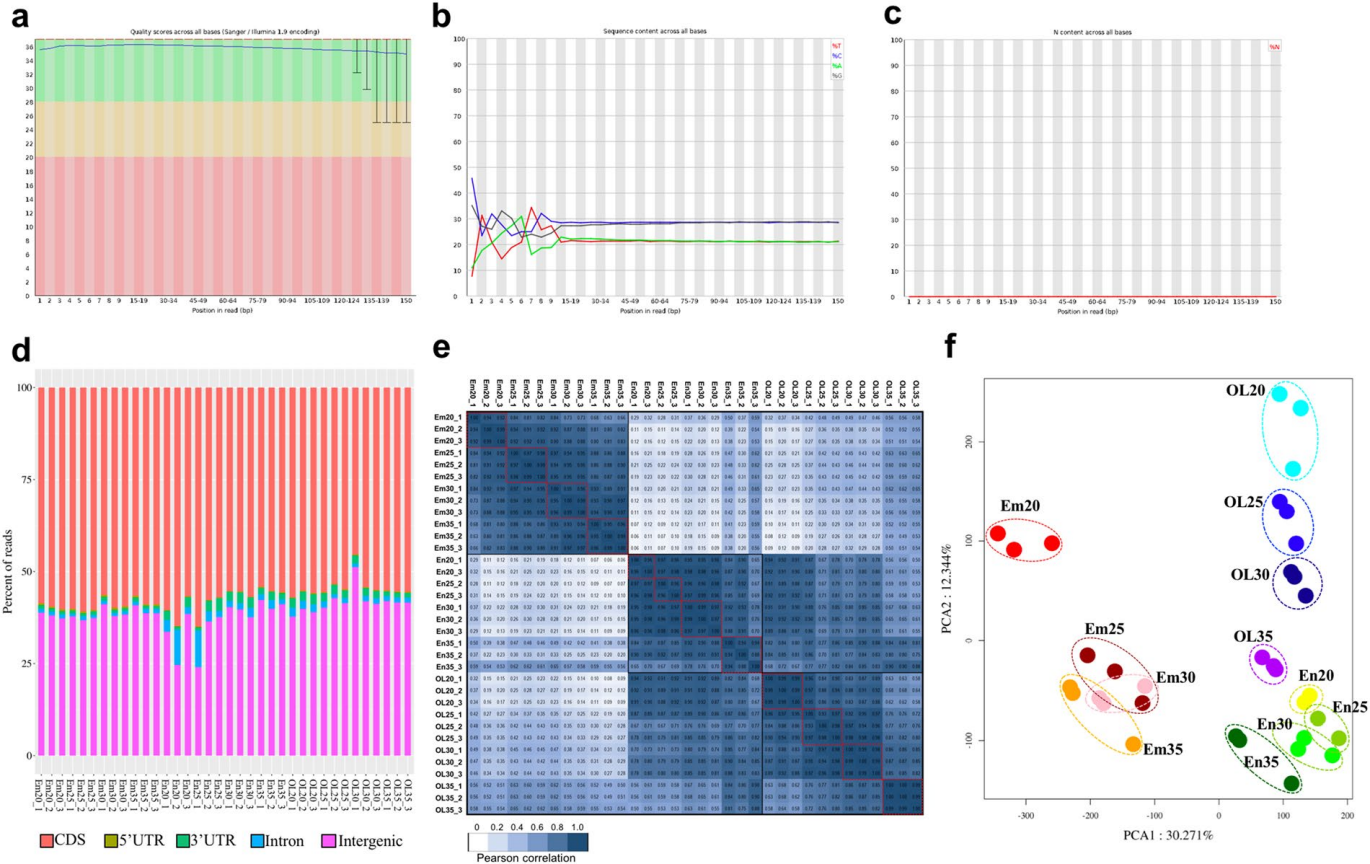
Quality control and clustering analysis of the RNA-seq data set. (**a**) Quality score of per position in the reads. (**b**) Sequence content per base of the clean data. (**c**) N content per base of the clean data. (**d**) The percent of reads mapped to coding sequences (CDS), 5′untranslated regions (5′UTR), 3′untranslated regions (3′UTR), introns and intergenic regions for each RNA-seq sample. (**e**) Hierarchical clustering analysis. (**f**) Primary component analysis (PCA) of the RNA-seq samples. The analyzed results of Em20_1 sample are shown as a representative in the (**a–c**).

The RNA-seq experiments obtained high sequencing quality scores and high clean read ratios. The Q30 scores range from 87.67% to 95.49% and the clean read ratios ranges from 97.18% to 99.37% (Supplementary Table S1; Fig. 2a–c; samples’ metadata in Supplementary Table S2). The GC content of all RNA-seq samples was relatively stable, varying from 48.95% to 57.89% (Supplementary Table S1). The percentage of RNA-seq reads mapped to different parts of coding regions demonstrate that a large portion (from 45.4% to 60.84%) of the reads were mapped to coding sequence (CDS) with a small fraction of the reads mapped to 5′ and 3′ untranslated regions (0.12% to 0.54% for 5′UTR and 0.77% to 2.94% for 3′UTR, respectively) for most of the RNA-seq samples (Fig. 2d). However, two samples, En20-2 and En25-1, might be skewed compared to the other samples, as they contained 9.51% and 9.92% percentage of reads, respectively, mapped to intronic regions, indicating DNA contamination (Fig. 2d). Consistent with this result, En20-2 and En25-1 RNA samples indeed had lower A260/230 scores (1.51 and 1.32, respectively; Table 1) and lower GC contents (49.45% and 48.95%, respectively; Supplementary Table S1). The lowered A260/230 and GC content of En20-2 and En25-1 samples also indicate DNA contamination in these samples. In addition, sample OL30-1 had a higher percentage of reads mapped to intergenic regions (Fig. 2d). Taken together with its low mapping rate but relatively acceptable uniquely mapped rate and the global expression pattern correlated well with the corresponding replicates, we think OL30-1 can be acceptable for gene expression analysis (Fig. 2e,f; Supplementary Table S1).

**Fig. 3:**
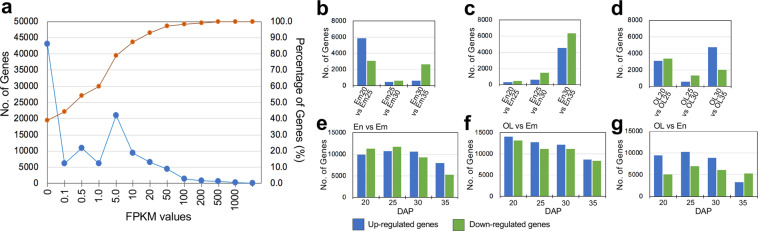
Gene expression of the RNA-seq samples (**a**) and differential expression analyses between the stages (**b**–**d**) or the tissue types (**e**–**g**). (**a**) The distribution of gene expression values (in FPKM) using Em20_1 as a representative. (**b**–**d**) The number of DEGs identified between the stages from embryo (**b**), endosperm (**c**) and the outer layers (**d**). (**e**–**g**) The number of DEGs identified between the tissue types from the same stages.

Differential expression analysis and the expression values were calculated for the high-confidence geneIDs annotated in the wheat reference genome (Supplementary Table S1, Table S2). Among the 110,790 annotated genes, a large portion had expression values ranging from 1 to 100 in the FPKM unit for each RNA-seq sample (a representative FPKM distribution per sample shown in Fig. 3a), supporting FPKM > 0.5 as a reasonable threshold for the expressed genes. The number of expressed genes varied from 37,862 to 51,368 (Supplementary Table S4), consistent with previous reports of wheat seeds samples^43^. The results of correlation heatmap and principle component analysis (PCA) based on the expression between the replicates further support that En20-2 and En25-1 samples are deviated from the remaining RNA-seq samples (Supplementary Figure S1). Thus, En20-2 and En25-1 samples are excluded from the identification of differentially expressed genes (DEGs). The number of DEGs between each pair of RNA-seq samples are shown (Fig. 3, Supplementary Table S4). Also, when En20-2 and En25-1 were excluded, the remaining RNA-seq samples could be well separated in the correlation heatmap and PCA plot (Fig. 2e,f, respectively). In the PCA result, the principle components 1 (PC1) and 2 (PC2) explains 30.27% and 12.34% of the variations, respectively, and well corresponds to the tissue types and developmental stages. The PCA results show: (1) the three tissue types can be clearly separated; (2) the outer layer and embryo tissues exhibit greater changes in gene expression during the four stages compared to the endosperm tissues. Indeed, the number of DEGs between the three tissues support this finding, with at least over 5000 up- or down-regulated genes identified at each of the four stage (Fig. 3e–g). By contrast, the number of DEGs between different stages within a particular seed tissue are much less, ranging from several hundreds to a few thousands. For example, only a few hundred genes were up- or down-regulated in the comparison of En20-En25 and En25-En30, consistent with the relative stable transcriptomic status of the endosperm samples as indicated in the PCA results (Figs. 2f, 3). In brief, the results of PCA and differential expression analysis consistently reflect the transcriptomic differences between the seed tissues and stages, demonstrating that this RNA-seq dataset is of high quality to study the gene expression regulation in different seed tissues during developmental stages.

### Quality validation for the metabolomic samples

**Fig. 4:**
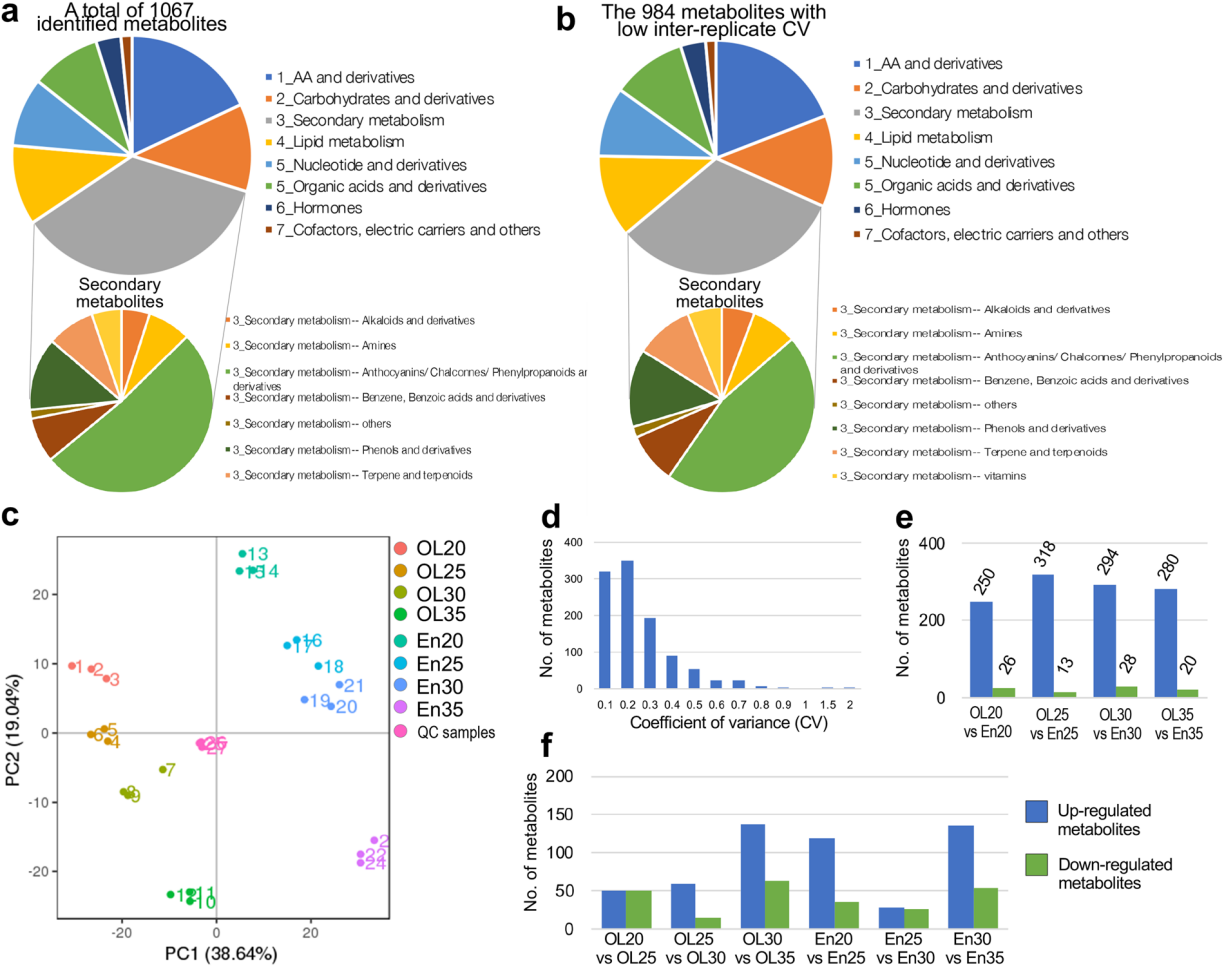
Characterization of the metabolomic data. (**a**) Classification of the 1,067 identified metabolites. (**b**) Classification of the 984 metabolites with low coefficient of variance values within the replicates. (**c**) PCA analysis of the metabolic samples. (**d**) The distribution of coefficient of variance values within the replicates for each metabolite (the three replicates of OL20 were used as a representative). (**e**) The number of differential metabolites between outer layer samples and endosperm samples at each of the four stages. (**f**) The number of differential metabolites between the stages within each the outer-layer or endosperm tissue.

The metabolomic analysis identified 1,067 known metabolites in the samples of wheat endosperm (En) and seed outer layers (OL), covering a diverse set of compounds, including amino acids, carbohydrates, lipids, nucleotides, organic acids and their derivatives and many metabolites belong to secondary metabolic pathways (Fig. 4a). By calculating the coefficient of variance of each metabolite within the three replicates, 984 metabolites^67,68^ with low CV values (<0.8) have been kept (CV distribution shown in Fig. 4d and Supplementary Fig. 2, meta-data for the metabolomic samples in Supplementary Table S2), with their metabolic categories given in Fig. 4b. PCA analysis well detected the metabolic differences among the samples, with PC1 and PC2 explaining 38.64% and 19.04% metabolic variations, respectively, and probably corresponding to the differences between the tissues and the stages (Fig. 4c). Partial least squares discriminant analysis (PLS-DA) was performed to identify the metabolites with differential abundance between the samples (Fig. 4e,f, Supplementary Table S5). Interestingly, around 250 to 300 metabolites were found to be up-regulated in the outer layer samples at each of the four stages (Fig. 4e), consistent with previous results that the outer layers of wheat seeds have more metabolites with higher abundance^22^.

## Supplementary information

Supplementary information 1

Medium sized supplementary Tables

## Data Availability

No custom code was generated for this work.
