# Supplementary material for: A multi-omic resource of wheat seed tissues for nutrient deposition and improvement for human health
Source: Sci Data. 2023 May 10;10:269. doi: 10.1038/s41597-023-02133-y (PMC10172328; doi:10.1038/s41597-023-02133-y)
Supplement: Supplementary file 1 — Supplementary information 1 [file 41597_2023_2133_MOESM1_ESM.docx]

**Supplementary Information for**

**A multi-omic resource of wheat seed tissues for nutrient deposition and improvement for human health**

Jingjing Zhi^1,#^, Jian Zeng^2,#^, Yaqiong Wang^1,#^, Hongyan Zhao^1^, Guoli Wang^1^, Jing Guo^2^, Yuesheng Wang^1^, Mingjie Chen^1^, Guangxiao Yang^1^, Guangyuan He^1^, Xiaoyuan Chen^2,*^, Junli Chang^1,*^, Yin Li^1,*^

**Author Affiliations:**

1. The Genetic Engineering International Cooperation Base of Chinese Ministry of Science and Technology, The Key Laboratory of Molecular Biophysics of Chinese Ministry of Education, College of Life Science and Technology, Huazhong University of Science & Technology, Wuhan 430074, China
2. Guangdong Provincial Key Laboratory of Utilization and Conservation of Food and Medicinal Resources in Northern Region, Henry Fok School of Biology and Agriculture, Shaoguan University, Shaoguan, Guangdong 512005, China

# These authors contributed equally to this work.

* Corresponding Authors: Xiaoyuan Chen (chenxy2@163.com), Junli Chang (cjl@hust.edu.cn), Yin Li (yinli2021@hust.edu.cn)

**Supplementary Figure S1** – **The heatmap of expression correlation and PCA result for all thirty-six RNA-seq samples**.

**Supplementary Figure S2** – **The distribution of CV of the metabolite abundance in each of the metabolomic samples**.

**Supplementary Table S1** – **The statistical summary of the RNA-seq samples.** Mapping rate is defined as the percentage of mapped reads out of the number of clean reads, while uniquely mapped ratio is defined as the percentage of uniquely mapped reads out of the number mapped reads. (Provided in a separate EXCEL file)

**Supplementary Table S2** – **Metadata of the wheat samples subject to transcriptomic and metabolomic analyses.** (Provided in a separate EXCEL file)

**Supplementary Table S3** – **The five key MS parameters (*i.e.*, Q1, Q3, RT, DP (de-clustering potential) and CE (collision energy)) of ten representative metabolites.** (Provided in this supplementary information file)

**Supplementary Table S4** – **Pair-wise comparison of the RNA-seq samples for differential expression analysis and the number of up- and down-regulated genes**.

**Supplementary Table S5** – **Pair-wise comparison of the metabolomic samples for differential metabolite analysis and the number of up- and down-regulated metabolites**.


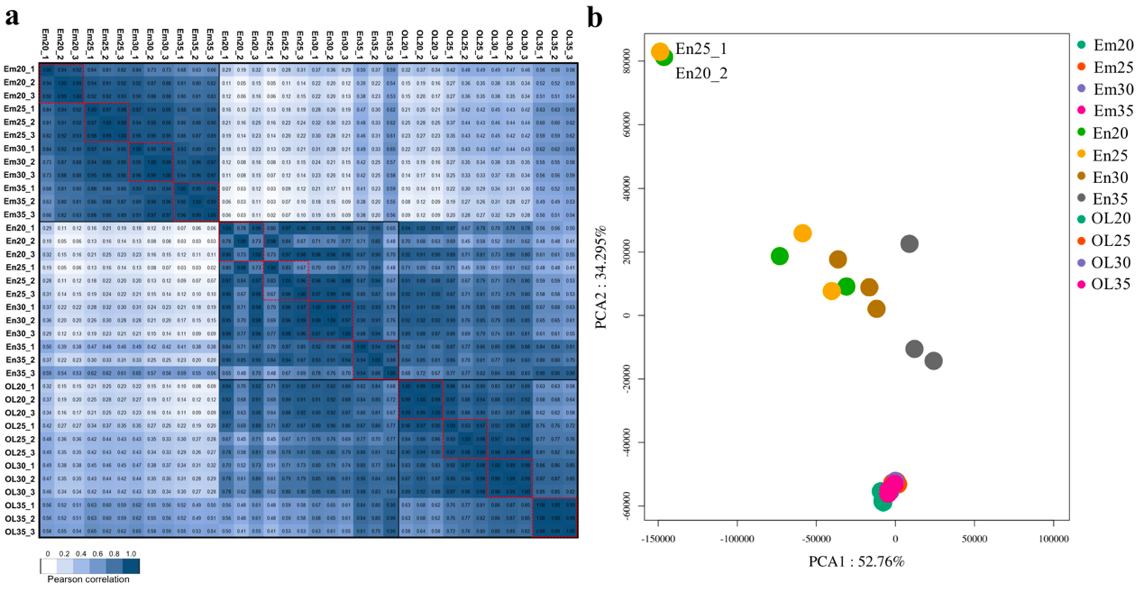


**Supplementary Figure S1** The heatmap of expression correlation (a) and PCA result for all thirty-six RNA-seq samples (b). Both of the heatmap of expression correlation and the PCA plot demonstrate that En20_2 and En25_1 are outlier RNA-seq samples deviated from the remaining replicates.


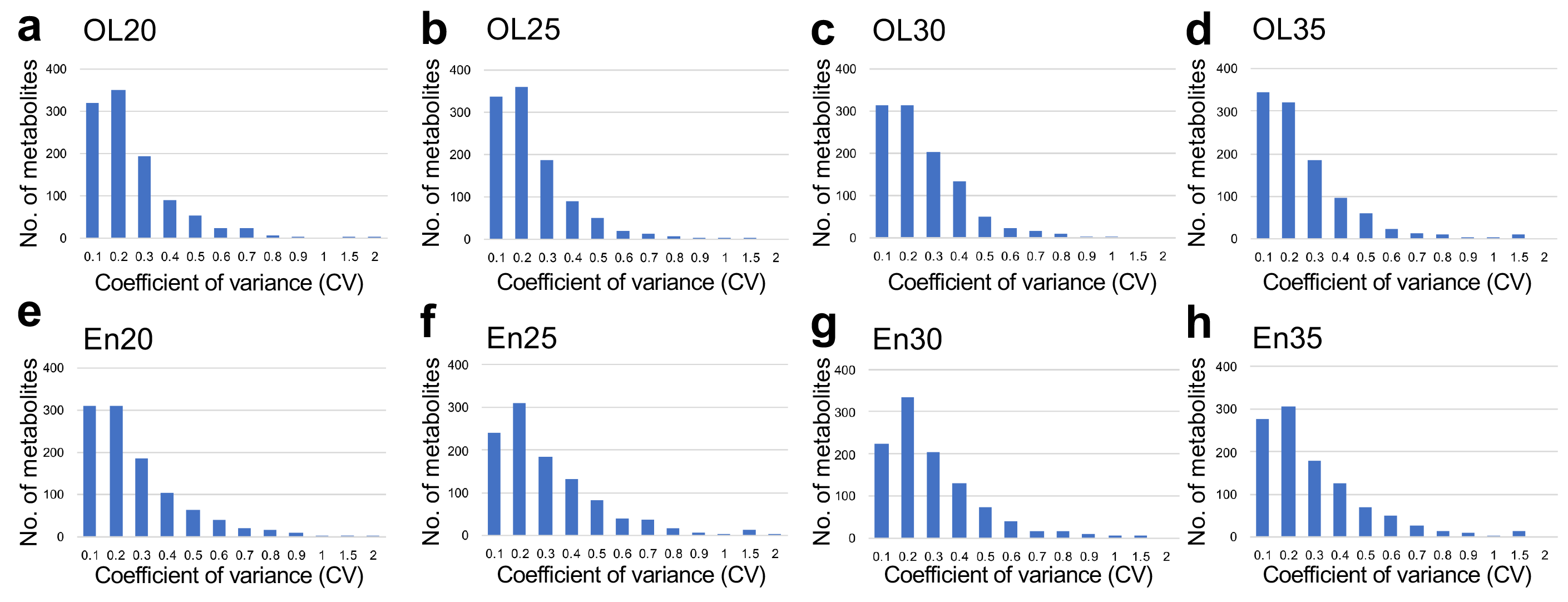


**Supplementary Figure S2** The distribution of CV of the metabolite abundance in each of the metabolomic samples. CV, coefficient of variance.

**Supplementary Table S3** The five key MS parameters (*i.e*., Q1, Q3, RT, DP (de-clustering potential) and CE (collision energy)) of ten representative metabolites.

| **Q1** | **Q3** | **RT** | **DP** | **CE** | **Name** | **Formula** | **Molecular Weight** | **RT [min]** | **Metabolite Class** | **CAS No.** |
| --- | --- | --- | --- | --- | --- | --- | --- | --- | --- | --- |
| 175.120 | 70.060 | 0.79 | 80 | 30 | L-arginine | C6H14N4O2 | 174 | 0.79 | Amino Acid And Derivatives | "74-79-3" |
| 122.000 | 76.000 | 0.88 | 30 | 19 | L-cysteine | C3H7NO2S | 121 | 0.88 | Amino Acid And Derivatives | "52-90-4" |
| 176.100 | 113.000 | 0.89 | 20 | 20 | L-Citrulline | C6H13N3O3 | 175 | 0.89 | Amino Acid And Derivatives | "372-75-8" |
| 421.076 | 241.012 | 0.96 | -100 | -30 | Trehalose 6-phosphate | C12H23O14P | 422 | 0.96 | Carbohydrates And Its Derivatives | "4484-88-2" |
| 181.072 | 59.015 | 0.88 | -100 | -40 | Mannitol | C6H14O6 | 182 | 0.88 | Carbohydrates And Its Derivatives | "69-65-8" |
| 261.040 | 78.960 | 0.86 | -60 | -30 | Sorbitol 6-phosphate | C6H15O9P | 262 | 0.86 | Carbohydrates And Its Derivatives | "20479-58-7" |
| 179.056 | 59.015 | 0.94 | -100 | -35 | D-Glucose | C6H12O6 | 180 | 0.94 | Carbohydrates And Its Derivatives | "50-99-7" |
| 341.000 | 119.000 | 0.98 | -80 | -20 | Sucrose | C12H22O11 | 342 | 0.98 | Carbohydrates And Its Derivatives | "57-50-1" |
| 117.000 | 73.100 | 1.94 | -30 | -13 | Succinic acid | C4H6O4 | 118 | 1.94 | Tca Cycle | "110-15-6" |
| 191.010 | 111.010 | 1.52 | -30 | -16 | Citric acid | C6H8O7 | 192 | 1.52 | Tca Cycle | "77-92-9" |

**Supplementary Table S4** Pair-wise comparison of the RNA-seq samples for differential expression analysis and the number of up- and down-regulated genes. DEGs, differentially expressed genes.

| **Comparison** | **Total No.**  **of DEGs** | **Up-regulated**  **genes** | **Down-regulated**  **genes** |
| --- | --- | --- | --- |
| Em20 vs Em25 | 8905 | 5823 | 3082 |
| Em25 vs Em30 | 1042 | 446 | 596 |
| Em30 vs Em35 | 3258 | 617 | 2641 |
| Em20 vs Em30 | 11636 | 6598 | 5038 |
| Em20 vs Em35 | 15295 | 7851 | 7444 |
| Em25 vs Em35 | 6993 | 2322 | 4671 |
| En20 vs En25 | 798 | 321 | 477 |
| En25 vs En30 | 2091 | 614 | 1477 |
| En30vs En35 | 10883 | 4544 | 6339 |
| En20 vs En30 | 2159 | 629 | 1530 |
| En20 vs En35 | 13626 | 5454 | 8172 |
| En25 vs En35 | 16670 | 6804 | 9866 |
| OL20 vs OL25 | 6452 | 3106 | 3346 |
| OL25 vs OL30 | 1925 | 608 | 1317 |
| OL30 vs OL35 | 6773 | 4726 | 2047 |
| OL20 vs OL30 | 9952 | 4836 | 5116 |
| OL20 vs OL35 | 17477 | 10025 | 7452 |
| OL25 vs OL35 | 11340 | 6803 | 4537 |
| En20 vs Em20 | 21147 | 9864 | 11283 |
| En25 vs Em25 | 22407 | 10680 | 11727 |
| En30 vs Em30 | 19923 | 10585 | 9338 |
| En35 vs Em35 | 13309 | 8008 | 5301 |
| OL20 vs Em20 | 27233 | 14025 | 13208 |
| OL25 vs Em25 | 23845 | 12716 | 11129 |
| OL30 vs Em30 | 23318 | 12130 | 11188 |
| OL35 vs Em35 | 17094 | 8701 | 8393 |
| OL20 vs En20 | 14553 | 9430 | 5123 |
| OL25 vs En25 | 17254 | 10286 | 6968 |
| OL30 vs En30 | 14903 | 8838 | 6065 |
| OL35 vs En35 | 8571 | 3265 | 5306 |

**Supplementary Table S5** Pair-wise comparison of the metabolomic samples for differential metabolite analysis and the number of up- and down-regulated metabolites.

| **Comparison** | **Total No. of differential metabolites** | **Up-regulated**  **metabolites** | **Down-regulated**  **metabolites** |
| --- | --- | --- | --- |
| OL20 vs En20 | 276 | 250 | 26 |
| OL25 vs En25 | 331 | 318 | 13 |
| OL30 vs En30 | 322 | 294 | 28 |
| OL35 vs En35 | 300 | 280 | 20 |
| OL20 vs OL25 | 101 | 50 | 51 |
| OL20 vs OL30 | 171 | 116 | 55 |
| OL20 vs OL35 | 357 | 255 | 102 |
| OL25 vs OL30 | 73 | 59 | 14 |
| OL25 vs OL35 | 294 | 233 | 61 |
| OL30 vs OL35 | 201 | 137 | 64 |
| En20 vs En25 | 155 | 119 | 36 |
| En20 vs En30 | 231 | 164 | 67 |
| En20 vs En35 | 391 | 304 | 87 |
| En25 vs En30 | 53 | 28 | 25 |
| En25 vs En35 | 239 | 170 | 69 |
| En30 vs En35 | 190 | 136 | 54 |
